# Evolution of a Species-Specific Determinant within Human CRM1 that Regulates the Post-transcriptional Phases of HIV-1 Replication

**DOI:** 10.1371/journal.ppat.1002395

**Published:** 2011-11-17

**Authors:** Nathan M. Sherer, Chad M. Swanson, Stéphane Hué, Roland G. Roberts, Julien R. C. Bergeron, Michael H. Malim

**Affiliations:** 1 Department of Infectious Diseases, King's College London School of Medicine, London, United Kingdom; 2 MRC/UCL Centre for Medical Molecular Virology, Division of Infection and Immunity, University College London, London, United Kingdom; 3 Department of Medical and Molecular Genetics, King's College London School of Medicine, London, United Kingdom; 4 Department of Biochemistry and Molecular Biology, University of British Columbia, Vancouver, British Columbia, Canada; University of Geneva, Switzerland

## Abstract

The human immunodeficiency virus type-1 (HIV-1) Rev protein regulates the nuclear export of intron-containing viral RNAs by recruiting the CRM1 nuclear export receptor. Here, we employed a combination of functional and phylogenetic analyses to identify and characterize a species-specific determinant within human CRM1 (hCRM1) that largely overcomes established defects in murine cells to the post-transcriptional stages of the HIV-1 life cycle. hCRM1 expression in murine cells promotes the cytoplasmic accumulation of intron-containing viral RNAs, resulting in a substantial stimulation of the net production of infectious HIV-1 particles. These stimulatory effects require a novel surface-exposed element within HEAT repeats 9A and 10A, discrete from the binding cleft previously shown to engage Rev's leucine-rich nuclear export signal. Moreover, we show that this element is a unique feature of higher primate CRM1 proteins, and discuss how this sequence has evolved from a non-functional, ancestral sequence.

## Author Summary

HIV-1 requires multiple cellular co-factors to replicate, and non-human cells often carry species-specific variations in the genes encoding these co-factors that can prevent efficient replication. Here, the basis for murine cell-specific deficiencies in the late steps of HIV-1 replication is addressed. We show that differences between the mouse and human forms of the essential host protein CRM1, a protein required for the transport of macromolecules from the nucleus to the cytoplasm, underlie this problem. More precisely, murine CRM1, unlike its human counterpart, fails to fully support the function of the HIV-1 Rev protein, a factor necessary to transport viral RNAs to the cytoplasm. Key amino acid differences between the mouse/human CRM1 proteins are identified and computational analyses of divergent animal CRM1 proteins reveal a unique motif in higher primates likely acquired in response to ancient evolutionary pressures. This CRM1 element may represent a novel pathogen interaction site that evolved to evade prior infections, but is now contributing to the susceptibility of humans to HIV-1.

## Introduction

HIV-1 is unable to replicate in most non-human species due to species-specific differences in cellular factors that either inhibit or promote viral replication. In particular, non-human versions of the cellular restriction factors APOBEC3G, TRIM5α and tetherin/BST-2/CD317 can each potently inhibit HIV-1 replication because the HIV-1 encoded evasion strategies (*e.g.*, the viral Vif and Vpu proteins) are ineffective [1]. In other instances, HIV-1 does not replicate in certain species due to the lack of functional versions of cellular proteins necessary for completion of key aspects of the viral life cycle. Mice and other rodents represent notable examples and exhibit multiple cellular deficiencies in pathways required for efficient HIV-1 replication [2]. While these deficiencies have impeded the development of a small animal model with which to study HIV-1, murine cell lines have served as powerful tools for delineating important molecular attributes of species-specific HIV-1 co-factors, including the CD4 entry receptor [3], [4] and CCR5 co-receptor [5], as well as the cyclin T1 (CycT1/CCNT1) transcription co-factor [6], [7]. Significantly, the combined provision of human versions of CD4, co-receptor (CCR5 or CXCR4) and CycT1 to murine cell lines does not restore HIV-1 replication, largely reflecting additional deficiencies that affect post-transcriptional steps of the virus life cycle [8]–[10].

The HIV-1 genomic RNA (gRNA) serves as the viral mRNA encoding the Gag and Gag-Polymerase (Gag-Pol) structural proteins, the genetic substrate that is packaged by Gag into virions, and as an RNA scaffold that facilitates Gag-Gag interactions [11]. Moreover, the full-length gRNA also represents the viral pre-RNA, with the potential to undergo splicing in the nucleus to generate the entire repertoire of viral mRNAs. Therefore, full-length gRNA and a subset of partially spliced viral mRNAs retain functional introns; this represents a specific challenge for retroviruses because mRNAs containing introns are typically prevented from exiting the nucleus [12]. HIV-1 overcomes this barrier through the activity of its regulatory protein Rev. Rev is expressed from fully spliced viral mRNAs and targeted to the nucleus where it binds and multimerizes on a *cis*-acting HIV-1 RNA target called the Rev response element (RRE) found only within HIV-1 intron-containing mRNAs. Subsequently, Rev binds the cellular chromosomal region maintenance-1 (CRM1, also known as exportin-1/XPO-1) nuclear export receptor through its leucine-rich nuclear export signal (NES) thereby forming the viral ribonucleoprotein transport complex [13]. CRM1 is a member of the karyopherin-β family of nuclear transport receptors regulated by the small GTPase Ran, and engages NES-containing cargoes in the nucleus prior to transporting them through the nuclear pore complex for release into the cytoplasm [14]. CRM1-mediated nuclear export of gRNA therefore acts a switch to initiate the late stages of the viral life cycle, because the cytosolic accumulation of gRNA is necessary for the expression of the Gag and Gag-Pol proteins that ultimately assemble the virus capsid.

In mouse cells expressing hCycT1, the cytoplasmic abundance of HIV-1 gRNA and Gag protein synthesis are significantly reduced in comparison to human cells, and Gag is not efficiently targeted to plasma membrane assembly sites [6], [8], [9], [15]–[19]. HIV-1 particle production can be restored in mouse cells by either modulating Gag's amino-terminal matrix (MA) membrane targeting domain in ways that enhance membrane binding [15], [16], [18]–[20] or by reprogramming the nuclear export pathway used by Gag-encoding mRNAs without modifying the Gag coding region [18], [19], [21]. More specifically, we have demonstrated that replacing the RRE in intron-containing Gag mRNAs with four copies of the constitutive transport element (CTE) from Mason-Pfizer monkey virus (M-PMV) effectively restores efficient virus particle assembly in mouse cells [19]. The CTE mediates M-PMV gRNA nuclear export independently of CRM1 [22], leading us to propose that the nuclear export of RRE-encoding transcripts and Gag assembly competence are linked mechanistically [11], [18], [19].

Fusing HIV-1 infected mouse cells with human cells results in vastly improved levels of virus production, indicating that one or more human cellular factors function to complement these murine-specific defects [8], [9]. Mouse-human somatic cell hybrids were used to map the relevant gene(s) to human chromosome 2 (Ch2) [23], and recent work from Shida and colleagues studying rat cells identified species-specific activity in CRM1, a gene product of Chr2 [24], [25]. Here, we demonstrate that human CRM1 (hCRM1) rescues a defect in the nucleocytoplasmic transport of viral intron-containing RNAs, including the gRNA. The molecular determinant of CRM1 underlying this stimulatory activity is a defined cluster of amino acids on the outer face of hCRM1's ringed structure, discrete from the hydrophobic cleft that binds the leucine-rich Rev NES. Moreover, combined phylogenetic and functional analyses indicate that the stimulatory activity conferred by this element may have evolved exclusively in higher primates.

## Results

**Figure 1:**
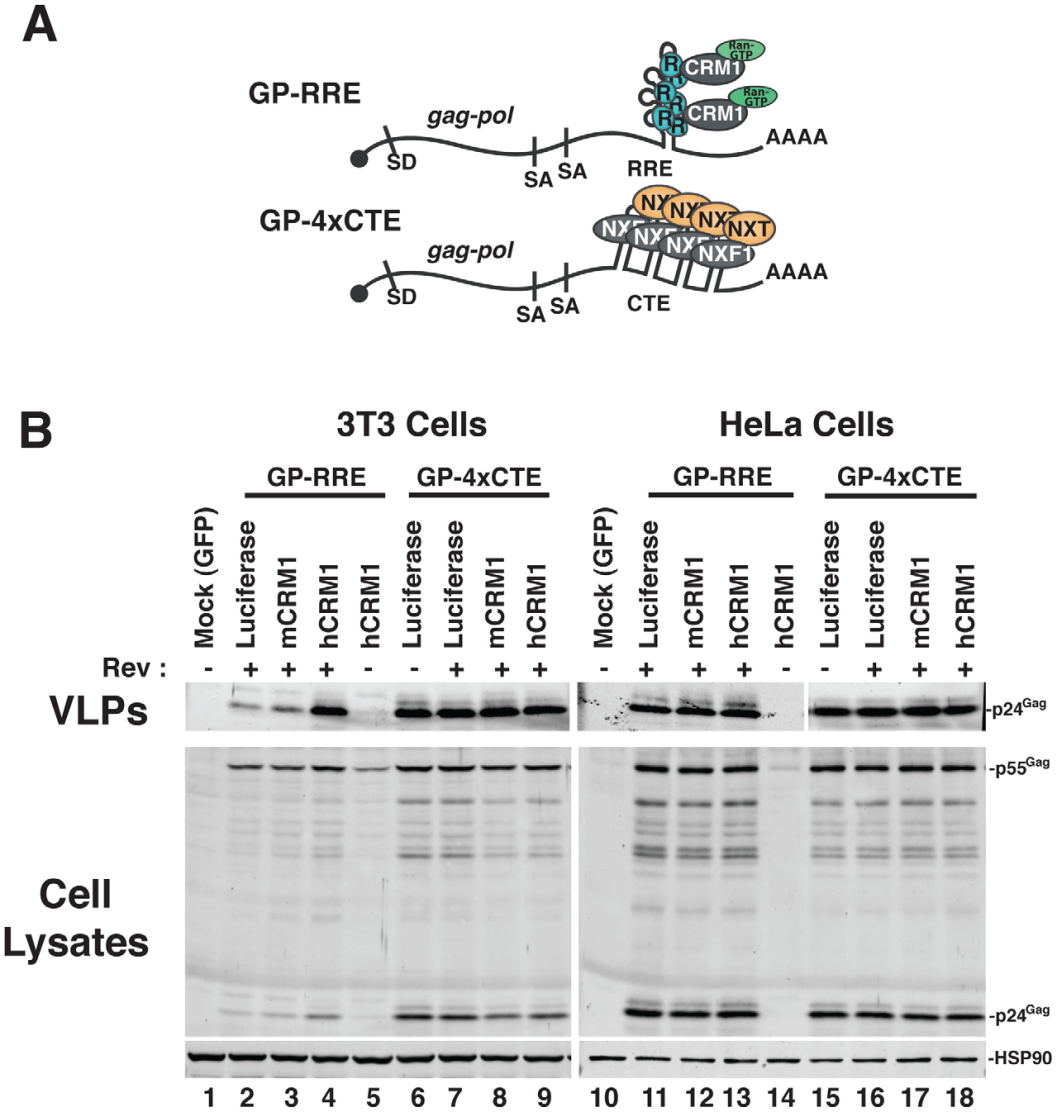
hCRM1 expression promotes HIV-1 VLP production in mouse cells. (A) Cartoon depicting the CMV-driven surrogate, intron-containing gRNA encoding Gag and Gag-Pol (GP) used in our screen. Rev-dependent, intron-containing transcripts (GP-RRE, top) carry a single Rev response element (RRE) forming 4 RNA stem loops predicted to accomodate 3 Rev (R) homodimers that recruite a maximum of two CRM1/Ran-GTP export complexes (based on [66]). Rev-independent GP transcripts (GP-4xCTE, bottom) carry four copies of the constitutive transport element (4xCTE) that binds to the NXF1/NXT1/2 heterodimer [54], [67]. With the exception of the export element, the GP-RRE and GP-4xCTE transcripts were identical and included the major splice donor (SD) and two native splice acceptors (SA's). Gag and Gag-Pol expression results exclusively from full-length, intron-containing transcripts. (B) 3T3 or HeLa cells were were transfected with pGP-RRE (1 µg) and 0.5 µg of plasmids encoding either luciferase, mCRM1 or hCRM1, with or without 0.25 µg pcRev as indicated. ∼48 h post-transfection, VLPs and cell lysates were harvested for immunoblot analysis using anti-p24^Gag^ or anti-HSP90 (loading control) antibodies.

We established murine NIH 3T3 cells as a platform to screen for viral and cellular determinants that affect HIV-1 post-transcriptional regulatory pathways [18], [19], [26]. To focus on post-transcriptional events, we engineered surrogate, intron-containing HIV-1 gRNA that encode Gag and Gag-Pol (derived from HIV-1_NL4-3_) and circumvent rodent-specific deficiencies affecting Tat-dependent transcriptional elongation [7], [27] and viral pre-RNA splicing [8], [28], [29]. To this end, we replaced the native HIV-1 promoter with the hCMV-IE promoter, that does not require HIV-1 Tat, and retained only the major 5′-splice donor and a subset of splice acceptors, thereby reducing the potential for oversplicing (Figure 1A). The *gag* and *pol* genes are located within the major intron and therefore Gag and Gag-Pol are expressed solely from full-length transcripts, for which nuclear export is differentially regulated by including either the HIV-1 RRE (GP-RRE; Figure 1A, top), that recruits Rev and CRM1, or four copies of the CTE from M-PMV (GP-4xCTE; Figure 1A, bottom), that recruits a heterodimer of NXF1 and NXT1/2. As previously demonstrated, Gag expression and virus-like particle (VLP) production were diminished for GP-RRE/Rev-dependent transcripts in 3T3 cells relative to human cells such as HeLa under identical transfection conditions (Figure 1B, compare lane 2 to lane 11) [19]. By contrast, CTE-dependent nuclear export in 3T3 cells resulted in a marked improvement to VLP production (Figure 1B, compare lane 6 to lane 2). Both nuclear export pathways were functionally equivalent in HeLa cells (Figure 1B, compare lane 11 to lane 15).

### hCRM1 rescues HIV-1 viron production in mouse cells

To address the hypothesis that defects in RRE-dependent virus production in murine cells reflect the lack of functional human versions of one or more factors, we screened a panel of human cDNAs encoding proteins with known functions in post-transcriptional regulatory pathways. These cDNAs were co-expressed with GP-RRE transcripts and Rev in 3T3 cells and we assayed for improvements to RRE/Rev-dependent Gag expression and VLP production [26]. In this screen, we identified hCRM1 as a factor whose expression led to an increase to VLP production relative to a luciferase control (Figure 1B, compare lane 4 to lane 2). hCRM1 effects on VLP production from GP-RRE transcripts were dependent on Rev expression (Figure 1B, compare lane 4 and lane 5) and were not exerted on GP-4xCTE transcripts that do not rely on Rev-dependent nuclear export (Figure 1B, compare lane 4 to lane 9). Subsequent experiments suggested that hCRM1 displayed substantially more activity in 3T3 cells than the murine version of CRM1 (mCRM1), indicating that the effect might reflect species-specific activity (Figure 1B, compare lane 4 to lane 3). By contrast, neither mCRM1 nor hCRM1 expression affected VLP production in human HeLa cells from either RRE/Rev-dependent or 4xCTE-dependent transcripts (Figure 1B, right panel). Taken together, these results highlighted mCRM1 as a candidate for the source of the defect to RRE/Rev-dependent HIV-1 virion production in 3T3 cells.

### hCRM1 effects on HIV-1 production are species-specific

**Figure 2 ppat-1002395-g002:**
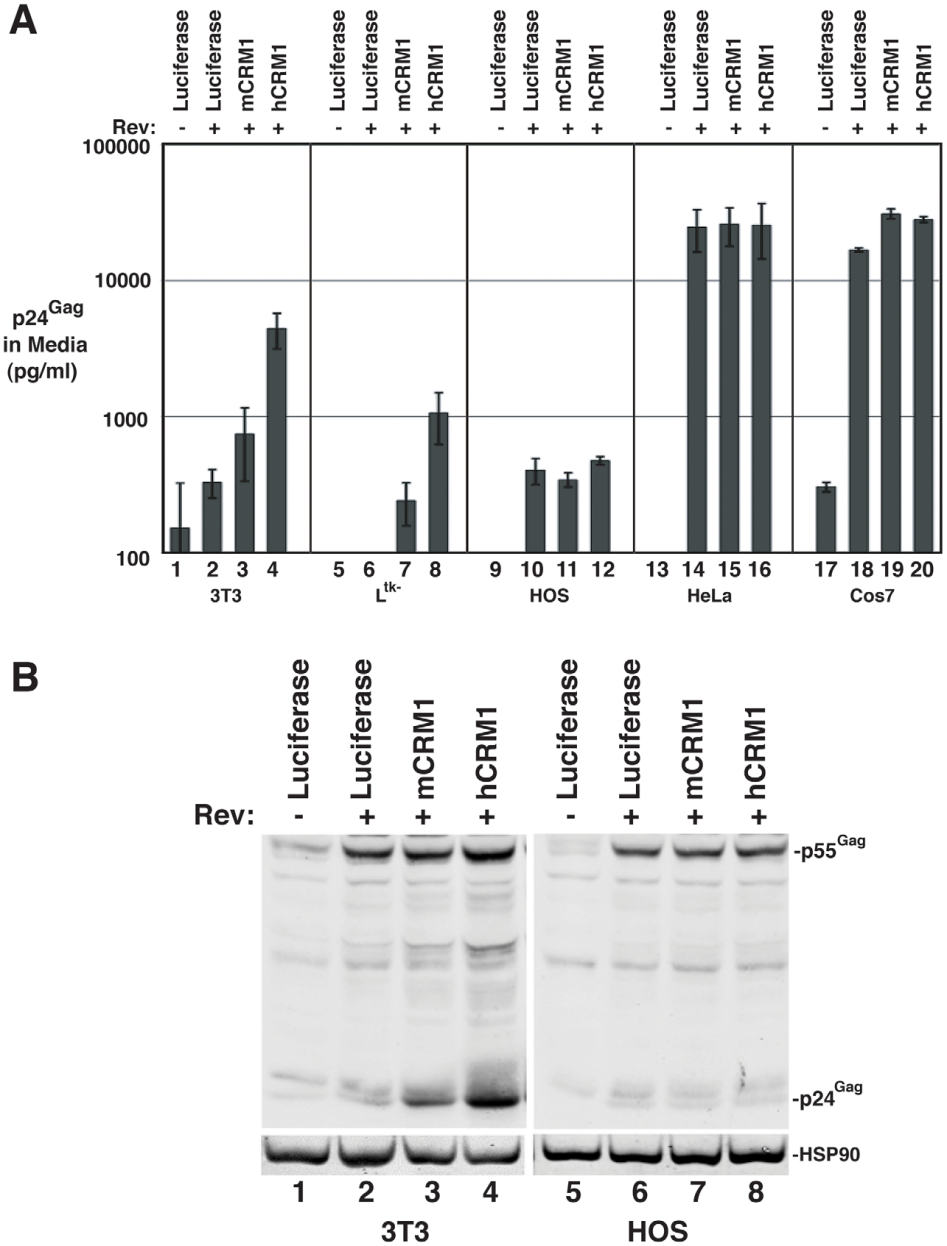
hCRM1 effects on HIV-1 VLP production are species-specific. A) The indicated cell lines were transfected as for Figure 1B. Supernatants were harvested at ∼48 h post-transfection and p24^Gag^ levels were measured by ELISA. Error bars represent the standard deviation for three independent transfections. In 3T3 cells, mCRM1 increased VLP production 2.9-fold (+/−1.4, *n* = 9, p = 0.0242 for mCRM1 vs. luciferase) and hCRM1 12.5-fold (+/−5.2 *n* = 9, p = 0.0045 for hCRM1 vs. Luc) relative to the luciferase control. B) Gag expression profiles for 3T3 and HOS cells transfected as for (A) and analyzed by immunoblot as for Figure 1B using antisera detecting p24^Gag^ and HSP90 (loading control).

We further assessed hCRM1 effects on RRE/Rev-dependent VLP production in a variety of cell lines, using an ELISA to quantify p24^Gag^ (capsid) levels in the cell supernatant at ∼48 h post-transfection (Figure 2). hCRM1 expression enhanced p24^Gag^ levels relative to mCRM1 in both 3T3 and murine L^tk-^ cells (Figure 2A, samples 1-8) but did not differentially affect VLP production in cells of human origin including human osteosarcoma (HOS) cells and HeLa cells (Figure 2A, samples 9-16), or in African green monkey Cos7 cells (Figure 2A, samples 17-20). Importantly, HOS cells exhibit low levels of Gag expression similar to 3T3 cells [20] and were not affected by hCRM1 expression (Figure 2A, compare samples 11 and 12, and Figure 2B, compare lanes 7 and 8), suggesting that hCRM1 responsiveness is not merely a corollary of low levels of Gag expression.

**Figure 3 ppat-1002395-g003:**
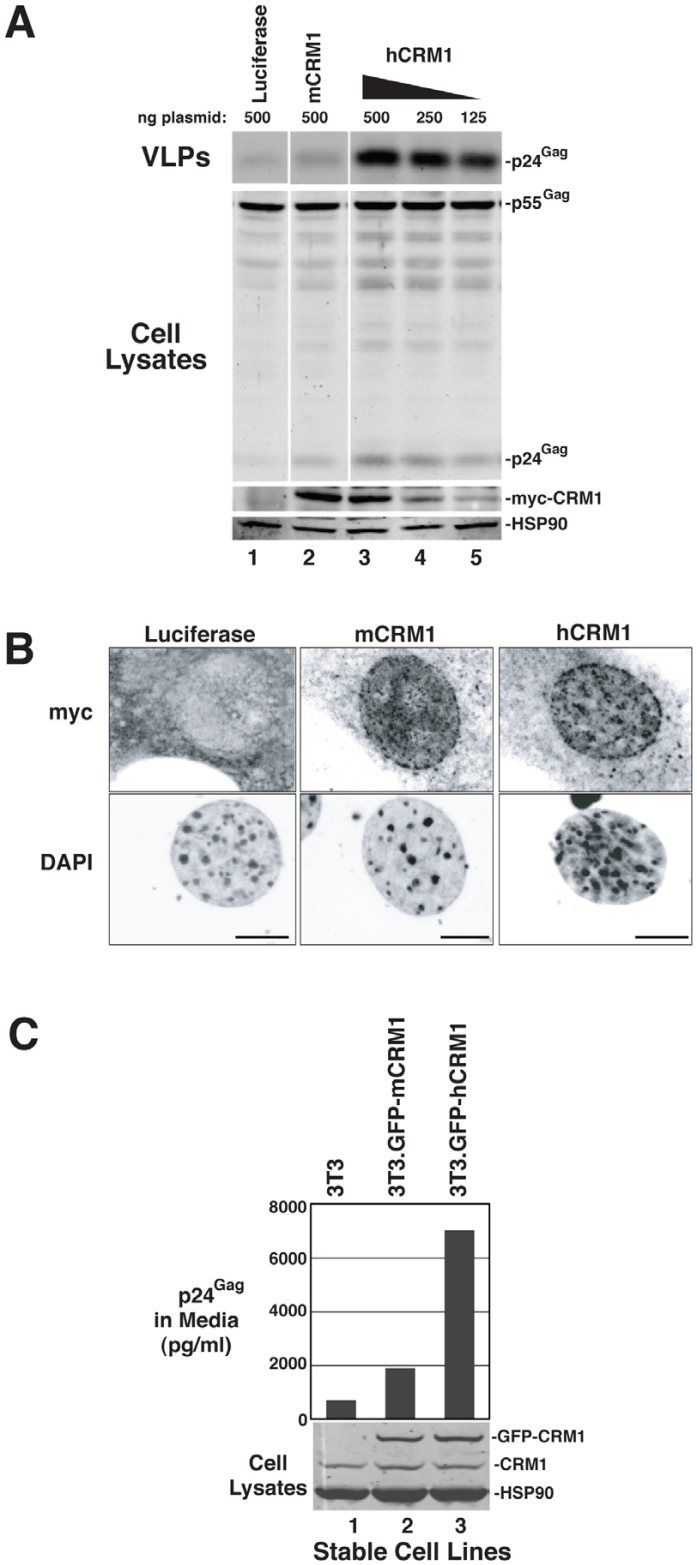
hCRM1 is more responsive to Rev than mCRM1. (A) 1 µg GP-RRE and 0.25 µg Rev plasmids were co-transfected into 3T3 cells with the indicated amounts of myc-tagged versions of luciferase, mCRM1 and hCRM1. Supernatants and cell lysates were harvested at ∼48 h post-transfection and analysed by immunoblot using anti-myc and anti-HSP90 (loading control) antibodies. myc-hCRM1 was 5.3-fold (+/−1.9, *n* = 3) more active than myc-mCRM1 in stimulating VLP production at 250 ng input of hCRM1 plasmid relative to 500 ng mCRM1 plasmid. (B) 3T3 cells transfected as for (A) were fixed on glass coverslips at ∼24 h post-transfection prior to visualization by indirect immunofluorescence using anti-myc antiserum and fluorescently conjugated secondary antibodies prior to confocal microscopy. Cell nuclei were visualized using DAPI. Images represent merged confocal *z*-slices covering ∼1 µm of the center of the cell. Size bars represent 5 µm. (C) 3T3 cells and 3T3 cells lines stably expressing GFP-mCRM1 or GFP-hCRM1 were transfected with 1 µg GP-RRE and 0.25 µg Rev plasmids and analyzed by p24^Gag^ ELISA and immunoblot. CRM1 species were detected using anti-CRM1 antibodies.

mCRM1 expression consistently resulted in slight increases to VLP production in murine cell lines relative to a luciferase control (*e.g.*, Figure 1B, Figure 2A), so that we directly compared the relative activities of mCRM1 and hCRM1. Varying amounts of myc epitope-tagged versions of these proteins were expressed with GP-RRE transcripts and Rev prior to detection by immunoblot using an anti-myc antiserum (Figure 3A). myc-hCRM1 was substantially more active than myc-mCRM1 in stimulating VLP production, even at lower levels of abundance (Figure 3A, compare lanes 3-5 to lane 2). The myc tag also allowed us to demonstrate by indirect immunofluorescence that both proteins exhibited similar intracellular distributions in 3T3 cells, localizing predominantly to the nucleus but with pronounced accumulation at the nuclear membrane (Figure 3B). To further test hCRM1 species-specificity, we established 3T3 cell lines that stably expressed GFP-tagged versions of mCRM1 (3T3.GFP-mCRM1) or hCRM1 (3T3.GFP-hCRM1). Compared to the parental cell line, VLP production was improved ∼4-fold for the cells expressing GFP-hCRM1 relative to GFP-mCRM1, despite similar levels of transgene expression relative to endogenous CRM1 (Figure 3C, compare lane 3 to lanes 1 and 2). In sum, these experiments demonstrated that hCRM1 exhibits species-specific activity compared to mCRM1 in enhancing HIV-1 particle production.

### hCRM1 induces HIV-1 gRNA nuclear export to stimulate the post-transcriptional stages of the viral life cycle in mouse cells

**Figure 4 ppat-1002395-g004:**
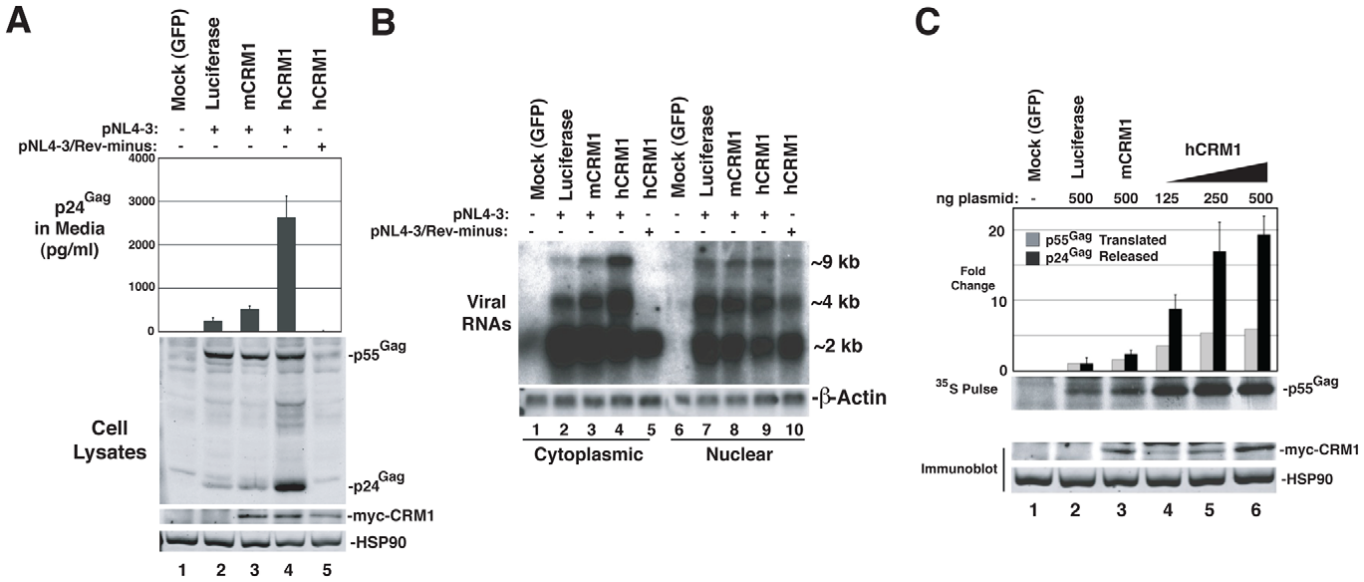
hCRM1 overcomes deficiencies in HIV-1 production in murine cells. (A) 3T3 cells were co-transfected with 1.0 ug HIV-1_NL4-3_ plasmid (lanes 2-4) or HIV-1_NL4-3_ Rev-minus plasmid (lane 5), 0.5 µg mCycT1-Y261C plasmid and either 0.5 µg mCRM1 plasmid (lane 3) or hCRM1 plasmid (lanes 4 and 5). Cells and supernatants were harvested at 48 h post-transfection and analyzed by immunoblot and p24^Gag^ ELISA. myc-CRM1 species were detected using anti-myc antiserum. myc-hCRM1 expression increased HIV-1 particle release relative to myc-mCRM1 6.4-fold (+/−1.7 *n* = 7) as measured by ELISA. (B) 3T3 cells were transfected as for (A) and processed for northern blot analysis ∼48 h post-transfection. Cytoplasmic (lanes 1-5) and nuclear (lanes 6-10) RNA fractions were probed with [^32^P]dCTP-labeled DNA probes complementary to HIV-1 mRNA or β-actin mRNA (loading control). The Rev-minus control also served as a cell fractionation control. The relative abundance of cytoplasmic ∼9 kb unspliced RNA (gRNA) was increased ∼4.0-fold (+/−0.95, *n* = 3) in the presence of hCRM1 relative to mCRM1. Nuclear gRNA levels increased 2.1-fold +/− 1.2 comparing hCRM1 to mCRM1. (C) 3T3 cells were transfected as for (A) with the indicated plasmids. Supernatants were harvested at ∼43 h post-transfection for analysis by p24^Gag^ ELISA (black bars) and proteins were radiolabeled using [^35^S]methionine/cysteine for 20 min at 37°C. Cells were lysed and Gag was immunoprecipitated, resolved by SDS-PAGE, transferred to nitrocellulose and visualized using autoradiography. [^35^S]-labelled Gag was quantified using a phosphoimager. Fold changes to Gag levels are shown relative to the luciferase control. The pulse data is representative of 3 independent experiments and myc-hCRM1 expression enhanced the rate of Gag translation ∼3.6-fold (+/−1.2, *n* = 3) relative to myc-mCRM1 at 500 ng of input plasmid. A duplicate set of samples, shown below, were subjected to immunoblot analysis in order to detect myc-CRM1 species and HSP90. Error bars represent the standard deviation for three independent transfections.

To evaluate the functional consequences of hCRM1 expression on the individual post-transcriptional stages of the HIV-1 life cycle, we assessed hCRM1 effects in the context of the full-length HIV-1_NL4-3_ provirus (Figure 4). To ensure efficient Tat-dependent transcription from the HIV-1 promoter, we co-expressed a previously described version of murine CycT1 (tyrosine-261 changed to cysteine; Y261C) that is fully Tat-responsive in mouse cells [6], [27]. Consistent with the GP-RRE system, myc-hCRM1 expression increased HIV-1 particle release ∼6-fold relative to myc-mCRM1 as measured by ELISA (Figure 4A, compare lanes 3 and 4). myc-hCRM1 did not affect a Rev-deficient (NL4-3/Rev-minus) provirus, confirming that these effects were Rev-dependent (Figure 4A, lane 5). We also tested if these viruses were infectious by harvesting cell supernatants at 48 h post-transfection and adding them to TZM reporter cells (Figure S1). The combined expression of mCycT1-Y261C and myc-hCRM1 resulted in a ∼100-fold increase in infectious virus production from 3T3 cells relative to the expression of mCycT1-Y261C alone (Figure S1A, compare lanes 2 and 5) and, when normalized for levels of input p24^Gag^, this virus exhibited comparable infectivity to viruses harvested from HeLa cells (Figure S1B).

To test the effects of hCRM1 expression on HIV-1 RNA abundance in the cytoplasm, we performed northern blotting on samples from an experiment identical to that in Figure 4A, using a probe that detects the full repertoire of HIV-1 mRNAs that includes ∼9 kb unspliced, ∼4 kb partially-spliced and ∼2 kb fully-spliced transcripts. The intron-containing ∼9 kb and ∼4 kb transcripts harbor the RRE and require Rev for their nuclear export while the accumulation of ∼2 kb transcripts in the cytoplasm is independent of Rev activity. The intron-containing RNAs accumulated to low levels in the cytoplasm of 3T3 cells expressing wild-type provirus and, as anticipated, were absent from the cytoplasm in cells expressing a Rev-minus mutant (Figure 4B, compare lanes 2 and 5). The relative abundance of cytoplasmic ∼9 kb unspliced RNA (gRNA) was increased ∼4-fold by hCRM1 relative to mCRM1 (Figure 4B, compare lane 3 to lane 4).

We next directly compared hCRM1 effects on Gag synthesis rates and virus particle production. myc-hCRM1 expression led to an enhanced rate of Gag translation relative to myc-mCRM1 at all levels of input plasmid (Figure 4C) as measured by metabolic labeling, correlating well with the observed increases to gRNA levels in the cytoplasm. Interestingly, relative effects on net virus particle release as measured by p24^Gag^ ELISA for these conditions were ∼3-fold higher than the increase in translation rate (Figure 4C, compare lanes 3 and 6, black bars). Taken together, the results presented in Figure 4 demonstrated that the ectopic expression of hCRM1 in murine cells increases the cytosolic abundance gRNA, resulting in improved Gag expression and a more pronounced boost to the efficiency of virus particle production.

### hCRM1 enhances matrix-dependent Gag membrane targeting in mouse cells

**Figure 5:**
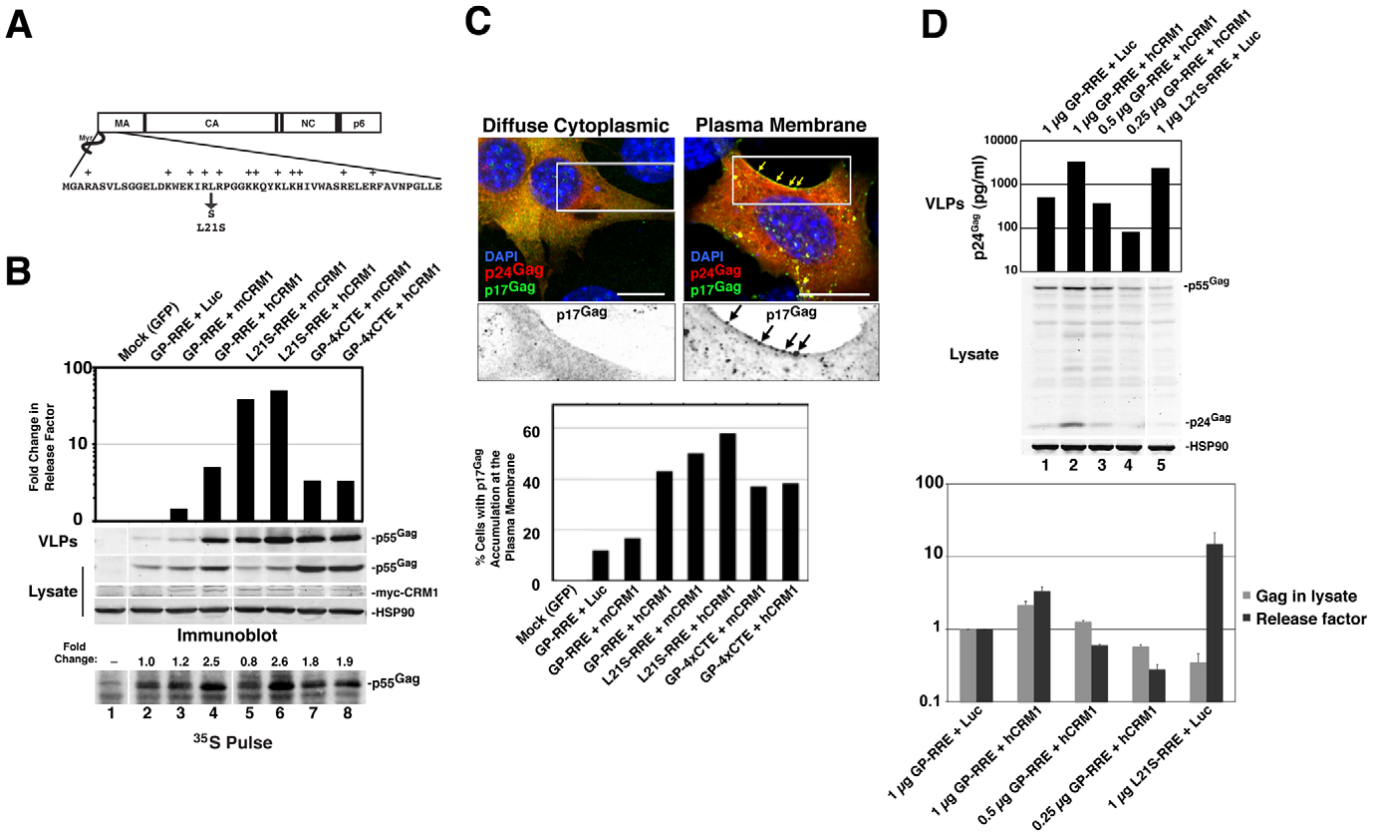
hCRM1 expression in mouse cells improves Gag assembly efficiency and trafficking to the plasma membrane. (A) Depiction of the Gag MA domain showing the amino-terminal myristoyl group and basic patch (basic residues are highlighted with a “+”). Replacement of leucine-21 with serine results in a mutant that constitutively targets the plasma membrane in 3T3 cells [18]. (B) 3T3 cells were transfected as for Figure 1B with the indicated Gag expression constructs. To prevent the proteolytic processing of Gag, the protease inhibitor saquinavir was added to a concentration of 1 µM at 24 h post-transfection. p55^Gag^, CRM1 and HSP90 were detected by immunoblot and Gag assembly efficiency was measured based on a “release factor”: the ratio of VLP-associated p55^Gag^ to cell-associated p55^Gag^. Values represent the fold change in release factor relative to the luciferase control (lane 2). The data is representative of 3 independent experiments. Shown at bottom, cells from an identical experiment were metabolically labeled for 15 minutes with [^35^S]methionine/cysteine ∼43 h post-transfection and Gag was detected as described for Figure 3C. Fold changes to Gag levels are relative to the luciferase control (gray bar, lane 2). hCRM1 expression enhanced the assembly efficiency of wild-type, Rev-dependent Gag (GP-RRE) relative to mCRM1 4.6-fold (+/-1.8, *n* = 5) corresponding to a increased Gag translation rate of 1.9-fold (+/−0.2, *n* = 3). (C) 3T3 cells were plated on glass coverslips, transfected as for (B) using the indicated Gag expression constructs and fixed at ∼30 h post-transfection prior to immunostaining with p24^Gag-^(red) and p17^Gag^-(green) specific antibodies that preferentially recognize immature p55^Gag^ and processed, mature p17^Gag^, respectively, followed by fluorescently conjugated secondary antibodies (upper panel). Cell nuclei were visualized using DAPI. Images represent a merger of 4 confocal *z*-slices to cover ∼1 µm in the *z*-dimension at the center of the cell, size bars represent 10 µm. 200 CA-positive (red) cells for each condition were scored blindly for evidence of MA (green) accumulation at the plasma membrane (lower panel). (D) 3T3 cells were transfected with 0.25 µg Rev and 0.5 µg hCRM1 or luciferase plasmids plus decreasing amounts of GP-RRE plasmid as indicated. Each transfection mix was normalized to 1.75 µg of total plasmid by adding additional luciferase plasmid as required. VLP levels were assessed using a p24^Gag^ ELISA and intracellular Gag and HSP90 were visualized by immunoblot (upper panels). Intracellular Gag levels were quantified and a release factor was calculated based on the ratio of released p24^Gag^ to intracellular Gag (lower panel). Error bars represent the standard deviation from three identical experiments.

In mouse cells, it is well-established that virus particle assembly is enhanced by modifications of Gag amino-terminal matrix domain (MA) that enhance Gag-membrane association [15], [16], [18]–[20]. MA encodes a bipartite plasma membrane targeting signal consisting of a hydrophobic myristoylation that modifies the amino-terminal glycine residue and a patch of basic amino acids distributed between amino acids 15 and 33 (Figure 5A) [30]. Gag membrane targeting is thought to be regulated by a myristoyl switch mechanism wherein the myristoyl group is sequestered within the MA globular head domain unless exposed in response to stimuli including Gag-Gag interactions and binding to the plasma membrane resident phosphoinositide PI(4,5)P_2_ [31], [32]. We recently described a Gag mutant carrying a single change to a non-charged amino acid, leucine-21 to serine (L21S) (highlighted in Figure 5A), that dramatically improves Gag assembly efficiency in murine cells, likely by circumventing the myristoyl switch mechanism in order to constitutively target Gag to the plasma membrane [18], [33].

To test if hCRM1 effects on Gag assembly in 3T3 cells are MA-dependent, we expressed Gag from GP-RRE subgenomic transcripts encoding wild-type Gag and Gag-L21S with myc-mCRM1 or myc-hCRM1 and measured virus assembly efficiency by calculating a “release factor” representing the ratio of released Gag to cell-associated Gag at 48 h post-transfection (Figure 5B). These experiments were performed under conditions where the viral protease was inactivated so that Gag levels could be measured by quantitative immunoblot as a discrete, uncleaved 55 kDa species. hCRM1 expression significantly enhanced the assembly efficiency of wild-type, Rev-dependent Gag (GP-RRE) relative to mCRM1 almost 5-fold, corresponding to an increased Gag translation rate of ∼ 2-fold (Figure 5B, compare lane 3 to lane 4). Moreover, these effects were CRM1-dose dependent (Figure S2). By contrast, hCRM1 had relatively little impact on the assembly efficiency of the Gag-L21S mutant that constitutively targets the plasma membrane [18] (Figure 5B, compare lanes 5 and 6). Overall VLP output of Gag-L21S increased ∼2-fold in the presence of hCRM1, corresponding not to better assembly efficiency but to improvements in Gag synthesis rates (Figure 5B, lower panel, compare lanes 5 and 6). Consistent with a rescue of Gag trafficking to virus assembly sites, single-cell visual analysis of Gag distribution under these conditions revealed a striking accumulation of Gag at the plasma membrane in 43% of cells expressing hCRM1 compared with 17% for mCRM1 (Figures 5C). In sum, hCRM1 expression promotes Gag's ability to traffic to the plasma membrane in mouse cells and efficiently assemble into virus particles.

The myristoyl switch in Gag is regulated by Gag multimerization, which is a cooperative process [32], [34]. Since hCRM1 exerted moderate effects on Gag expression in mouse cells but amplified effects on the production of virus particles (Figures 4C and 5B), we asked if these effects were cooperative and due to achieving a threshold level of intracellular Gag or, instead, reflected a second function for hCRM1 in modulating MA-dependent Gag membrane targeting. We titrated Gag expression plasmids in the presence of hCRM1 to achieve intracellular levels of Gag equivalent to that observed in the absence of hCRM1 expression. At comparable levels of Gag for either condition, we observed nearly identical levels of VLP production (Figure 5D top panel, compare lanes 1 and 3). Moreover, the magnitude of the release factor for virion production correlated with the intracellular abundance of Gag (Figure 5D, bottom panel). Therefore, hCRM1's effects on HIV-1 assembly in mouse cells can be attributed to its ability to enhance Gag expression (Figures 4C and 5B, lower panel), which is achieved through increasing the cytoplasmic level of gRNA.

### Identification of a functional domain within hCRM1 that stimulates HIV-l production in murine cells

**Figure 6:**
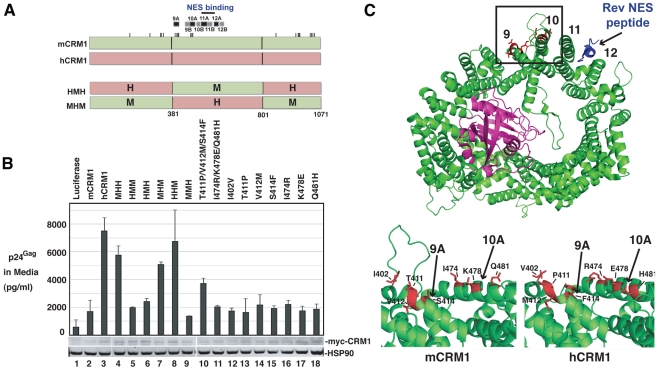
A species-specific determinant within hCRM1 is necessary for efficient HIV-1 virus production. (A) Depictions of mCRM1 (green), hCRM1 (red) and key mouse-human CRM1 chimerae. mCRM1 and hCRM1 exhibit 21 amino acid differences (ticks). The human-mouse-human (HMH) and mouse-human-mouse (MHM) chimeric proteins were constructed as shown. HEAT repeats 9-12 and the NES-binding cleft (blue line) are highlighted. (B) 3T3 cells were transfected with pGP-RRE, pcRev and plasmids encoding the indicated CRM1 species. VLP production was measured by p24^Gag^ ELISA at ∼48 h post-transfection and cell lysates were subjected to immunoblot using anti-myc and anti-HSP90 (loading control) antibodies. (C) Murine- and human-specific residues within HEAT repeat helices 9A and 10A are highlighted in red for mCRM1 (upper and bottom left panels, PDB ID: 3NBZ) [36] and hCRM1 (lower right panel, PDB ID: 3GB8) [35]. The loop shown only for mCRM1 including residue 402 (bottom left) was not resolved in the published hCRM1 structure. RanGTP (purple) and the Rev NES (blue) in complex with mCRM1 are as indicated. The figure was generated using PyMol.

To assess the relevance of individual domains of CRM1 to HIV-1 Rev function, we constructed eight myc-tagged CRM1 chimeras, alternating the mouse or human species identity of the amino-, central-, and carboxyl- portions of the protein as depicted in Figure 6A. When co-transfected with GP-RRE and Rev plasmids into 3T3 cells, we observed significant increases in VLP production whenever the central region (residues 381 to 800) of the chimera was derived from hCRM1 (Figure 6B, lanes 3,4,7 and 8). For instance, the mouse-human-mouse (MHM) chimera exhibited activity while the human-mouse-human (HMH) chimera did not, demonstrating that the activity within the central domain was both transferable and sufficient (Figure 6B, compare lanes 6 and 7).

CRM1 is a toroid-shaped molecule that is remarkably well-conserved throughout the animal kingdom, with murine and human versions of CRM1 differing at only 21 of 1071 amino acids (98% identity). CRM1 consists of 21 “HEAT” repeats; antiparallel alpha helices wherein the “A” helix faces outward on the convex face of the molecule and the “B” helix faces inward as depicted in Figure 6C. Recent structural work provides strong evidence for a model wherein Ran-GTP (in purple) binds to the inner surface of CRM1 and triggers allosteric changes in the hydrophobic NES binding pocket located within HEAT repeats 11 and 12, thereby promoting the binding of an NES-bearing cargo (Rev NES in blue) to form the trimeric CRM1/Ran/cargo export complex [35]-[38]. The mCRM1 and hCRM1 proteins differ at only seven positions within the central domain (Figure 6C, specific residues are highlighted in red), all of which are located on the outward-facing “A” helices of HEAT repeats 9A and 10A, with the exception of amino acid 402 that is situated on the loop just upstream of HEAT repeat 9A. HEAT repeats 9A and 10A form a contiguous surface “patch” that is over 20 Å away from the Rev NES binding site (Figure 6C, bottom panels and Figure S3, note; no density was observed for the loop including residue 402 in the hCRM1 crystal structure) and clearly does not interact with the Rev NES. Indeed, the amino acids that interact with Ran or the Rev NES [36], [37] are invarient between human and mouse. Single mouse-to-human amino acid substitutions in mCRM1, at each of the differing seven residues, were not sufficient to stimulate VLP production (Figure 6B, lanes 12 through 18). By contrast, a triple substitution (T411P/V412M/S414F) corresponding to the human configuration of amino acids in HEAT repeat helix 9A, consistently exerted a stimulatory effect (∼2-fold compared to mCRM1) on VLP production (Figure 6B, lane 10).

#### The functional domain in hCRM1 HEAT repeat 9A evolved specifically in higher primates

**Figure 7:**
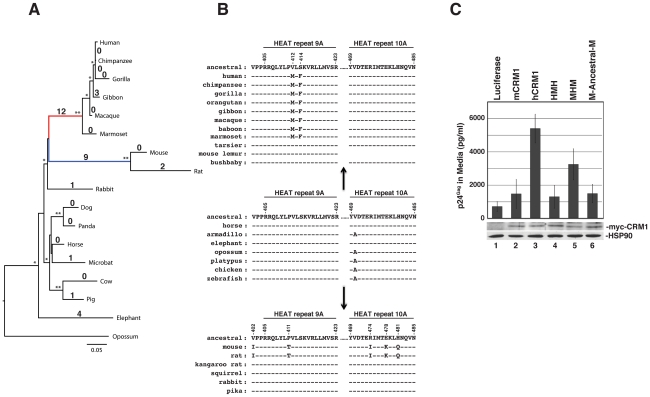
The hCRM1 stimulatory domain may be a specific adaptation of higher primates. (A) The phylogeny of 17 full-length CRM1 sequences was determined by maximum likelihood inference. Local support values >90% (one star) or  = 100% (two stars) are indicated on the branches. Branch lengths indicate the number of nucleotide substitutions per site. Values on the branches correspond to the number of amino acid changes compared to the reconstructed ancestral CRM1 sequence. The branches leading to higher primates (red) and murids (mice and rats) (blue) exhibited 12 and 9 changes, respectively, compared to the reconstructed ancestral sequence. (B) Amino acids within HEAT repeat helices 9A and 10A that differ between mCRM1 and hCRM1 (positions noted at top), and their changes from an “ancestral Euarchontoglires” consensus in this region. The A470V change appears to have occured in the evolution of the placental superorders, and the valine is present in all of the Euarchontoglires CRM1 sequences we analyzed. The sequence of HEAT repeat 10A was not available for the mouse lemur. (C) 3T3 cells were transfected with pGP-RRE, pcRev and plasmids encoding the indicated CRM1 proteins. VLP production and immunoblot analyses of cell lysates were performed as described for Figure 6B.

The clear dichotomy between human and mouse CRM1 in terms of supporting Rev function prompted us to consider its sequence evolution. We reconstructed the phylogeny of 17 full length mammalian CRM1 sequences using maximum likelihood inference (Figure 7A). Interestingly, the 21 amino acid differences between human and mouse CRM1 were due to two independent bursts of change, with 12 amino acidchanging substitutions in the primate lineage and 9 amino acid changing substitutions in the murid lineage of rodents that includes mice and rats. These two instances of diversification resulted in more amino acid changes than any other internal branch (or lineage) of the phylogeny (Figure 7A). 6/21 of these changes (28%) fell within HEAT repeat 9A or 10A while, combined, these two helices comprise only 3% of the protein's amino acids.

CRM1 overall, and HEAT repeat helices 9A and 10A in particular, is well conserved among representatives of the Laurasiatheria (horse), Afrotheria (elephant) and Xenarthra (armidillo) placental mammalian superorders (*e.g.*, Figure 7B). These two helices are also highly conserved in marsupials (opossum), monotremes (platypus), and other vertebrates such as the chicken and zebrafish, whose evolution from a common ancestor, combined, spans hundreds of millions of years. Further investigation of CRM1 changes in the primate lineage indicated that these amino acid substitutions occured abruptly in evolutionary time. For example, the tarsier, a primate, has a CRM1 amino acid sequence identical to horse CRM1, while the Simiiformes (new and old world monkeys) exhibit 12 amino acid differences (A191S, G334D, V412M, S414F, A869T, P961S, M972I, I974L, T1040I, Q1046D, L0152R, L1060F). All 12 of these changes occured at a similar time in mammalian evolution, when Simiiformes diverged from Tarsiiformes ∼80 million years ago [39]. They were subsequently fixed in all descending primates, as indicated by the presence of these amino acid alterations in all Simiiform CRM1 sequences that are currently available (Figure 7B). Because the amino acid changes in the central region of CRM1 are important for the function of CRM1 as a cofactor for HIV-1 RNA nuclear export (Figure 6B), the evolution of the two changes in Simiiformes HEAT repeat helix 9A (V412M and S414F) are highlighted in Figure 7B. A similar phenomenom occured in the rodent lineage, where all 9 changes (V284E, I337L, T346A, V402I, P411T, R474I, E478K, H481Q, E976D) occured in the lineage separating Muridae from Sciuridae (squirrels) and Heteromyidae (kangaroo rats) and are also not found in the lagomorphs rabbit and pika. The evolution of the five changes distributed in or near HEAT repeat helices 9A and 10A (V402I, P411T, R474I, E478K, and H481Q) in murids are highlighted in Figure 7B.

Recent analyses of several host antiviral restriction factors that interact with retroviral proteins have revealed that the sites of interaction have been subjected to positive, or Darwinian, selection: examples include APOBEC3G, APOBEC3F, TRIM5a and tetherin/BST-2/CD317 [40]–[43]. In particular, these coding elements are marked by a high ratio of non-synonymous (codon-altering; dN) changes relative to synonymous (silent; dS) changes within a coding region, resulting in dN/dS values > 1.0 [44]. The estimation of dN/dS across the entire gene by single likelihood ancestor counting method (SLAC) indicated that the evolution of CRM1 has been driven by very strong negative selection throughout mammalian evolution (mean dN/dS = 0.015 [95% confidence intervals: 0.011;0.019]), consistent with a strong sequence conservation among species. However, because dN/dS can vary for specific regions of a protein, we evaluated whether positive selection had occurred in individual domains of CRM1. Because primates and rodents are both members of the Euarchontoglires placental superorder, we compared human or mouse CRM1 to CRM1 from the horse, a member of the Laurasiatheria placental superorder, using a 30 bp sliding window dN/dS analysis (step-size of 3 bp). The human/horse comparison revealed two peaks of dN/dS >1 within HEAT repeats 9A and 21B (Figure S4A) while all other regions had a dN/dS < 1. A similar sliding window comparison between human and mouse CRM1 sequences demonstrated a dN/dS > 1 peak only within HEAT repeat 9A. However, all dN/dS values in both analyses were less than 2 and therefore did not completely rule out a scenario of neutral evolution, wherein dN/dS  =  1. In addition, codon-specific selection analyses using SLAC, fixed effect likelihood (FEL) and random effects likelihood (REL) failed to identify individual sites under continuous positive selection at the p<0.05 statistical level.

In order to test whether specific residues in the CRM1 HEAT repeat helices 9A or 10A were subjected to positive selection along the branches leading to the higher primates (red branch; Figure 7A) and to the murid rodents (blue branch; Figure 7B), we performed branch-site dN/dS analyses. These analyses, restricted to the HEAT repeat 9A plus residues 402-404, identified 2 positively selected sites on the branch leading to the higher primates at positions 412 and 414 as well as two positively selected sites on the branch leading to the rodents at positions 402 and 411 (Table S1). Branch-site analysis of HEAT repeat 10A identified all 3 rodent-specific changes (I474, K478 and Q481) as resulting from positive selection (Table S1). However, the model assuming positive selection along these branches did not provide a significantly better fit to the data compared to the neutral model (p>0.99) so that, again, an episode of neutral selection, or relaxed purifying selection, on these branches cannot be fully excluded. In sum, fixation of multiple changes in HEAT repeat 9A within the Simiiformes infraorder and HEAT repeat helices 9A and 10A in the Muridae family suggest two bursts of rapid amino acid alterations, resulting either from ancient selective pressures or an episodic relaxation of the negative constraints otherwise maintaining these particular regions.

Considering that both human and mouse CRM1 HEAT repeats 9A and 10A have substantially diverged from the ancestral sequence (Figure 7B), we sought to determine if the functional human CRM1 activity was due to its two amino acid changes (V412M, S414F) relative to the ancestral sequence or, alternatively, if the non-functional mouse CRM1 activity reflected its five changes (V402I, P411T, R474I, E478K, H481Q). We addressed this question by substituting the central domain of mCRM1 with the “ancestral” configuration of amino acids (Figure 7B, middle sequences) and measuring the ability of the ectopically expressed CRM1 chimeras to promote VLP production in 3T3 cells (Figure 7C). The activity of the M-ancestral-M CRM1 was equivalent to mCRM1, despite the fact that only residues 412 and 414 differed in this protein relative to the more active MHM chimera. Given that the mCRM1-T411P/V412M/S414F triple mutant exhibited some stimulatory activity (Figure 6B), we infer that the human, and not the ancestral, HEAT repeat helix 9A sequence is central to CRM1's capacity to serve as an effective co-factor for HIV-1 Rev function.

## Discussion

Here, we provide evidence that the human CRM1 protein contains a species-specific element required for efficient nucleocytoplasmic transport of Rev-dependent HIV-1 intron-containing RNAs and infectious HIV-1 production in murine cells (Figures 1-5). CRM1 is the major nuclear export receptor for cellular proteins, and maintains the nucleocytoplasmic partitioning of a broad array of cellular factors that regulate cell signaling and gene expression. These critical functions are emphasized by CRM1's high level of sequence conservation; for example, 98% amino acid identity is shared between human and mouse and 96% between human and fish (*Danio rerio*). Our results therefore present a striking example of how the evolution of subtle changes within an essential host protein, with no evidence of disturbing general cellular function, can have profound implications for the replication of an important human pathogen.

In keeping with our observations, the Shida lab previously demonstrated a synergistic effect for hCRM1 expression combined with human CycT1 expression in increasing HIV-1 production from rat macrophages [25], and recently reported a defect in rat CRM1 that specifically impacted HIV-1 assembly with effects that were largely independent of changes to cytoplasmic gRNA abundance, Gag levels or Gag trafficking to the plasma membrane [24]. By contrast, our work identifies ineffective Rev-mediated RNA nuclear export as the principal manifestation of murine CRM1 activity (Figure 4B) and we demonstrate that hCRM1 expression triggers a significant increase to cytoplasmic gRNA levels and intracellular Gag concentration in murine cells (*e.g.*, Figures 4 and 5). These increases likely underlie the observed stimulation of MA-dependent transport of Gag molecules to the plasma membrane (Figure 5), and are consistent with a model wherein cooperative, concentration-dependent Gag-Gag interactions regulate the efficiency of virus particle assembly [32], [34]. At a fundamental level, our data support the earlier Trono and Baltimore assertion that Rev-dependent nuclear export is deficient in mouse cells [17], and we conclude that the species-specific factor responsible for this defect is CRM1.

The ability of hCRM1 to stimulate HIV-1 production requires a species-specific configuration of amino acids on the convex surface of CRM1 within HEAT repeat helices 9A and 10A (Figure 6). Activity can be transferred from hCRM1 to mCRM1 by swapping the central domain, indicating that, in the context of either species' CRM1, this region is both necessary and sufficient for stimulating virus particle production in murine cells (Figure 6B, compare lanes 6 and 7). Remarkably, amino acids within HEAT repeats 9A and 10A are almost entirely conserved in all sequenced placental animals, with two notable sets of changes in the primate and rodent lineages (Figures 6C and 7B). Regarding hCRM1, we demonstrate that the insertion of the primate configuration of proline-411, methionine-412, phenylalanine-414 to the mouse protein (Figure 6B), as well as the removal of methionine-412 and phenylalanine-414 from the human central domain in the M-Ancestral-M chimera (Figure 7C) both impact on CRM1 activity, highlighting the biological significance of this surface-exposed element.

How might a CRM1 element impact Rev-dependent nucleocytoplasmic RNA transport, considering that the hydrophobic cleft that engages the Rev NES, located within HEAT repeats 11 and 12 (amino acids 514 to 575), is wholly conserved between hCRM1 and mCRM1 (Figure 6A), and throughout the animal kingdom? The implicated species-specific domain comprising HEAT repeat helices 9A and 10A is positioned more than 20 Å from the NES binding site (Figure S3). While significant, this distance might not exclude the formation of a secondary interface between CRM1 and one or more additional elements associated with Rev or the viral gRNA ribonucleoprotein complex. Indeed, CRM1 was reported to interact more strongly with Rev compared to a Rev NES peptide [45]. Moreover, protein footprinting analysis implied a secondary Rev/CRM1 interface, although these Rev-protected residues localize to CRM1 HEAT repeats 15 and 16 and not to HEAT repeat 9A/10A [46]. Importantly, several of the residues in HEAT repeats 9A and 10A that contrast between human and mouse CRM1, including proline-411, phenylalanine-414, arginine-474 and histidine-481, were implicated in CRM1's ability to recruit RanBP3 [47], a factor affecting CRM1's interaction with RanGTP and ability to bind to specific substrates [48]–[50]. Despite this finding, wild-type versions of human and rat CRM1 exhibit a similar capacity to engage RanBP3 [47] so the relevance of this particular interaction remains to be determined. Taken together, it will be important to further characterise how differences between the human, mouse and ancestral sequences of CRM1 influence its interaction with the Rev hexamer on the RRE as well as with other nuclear export co-factors.

Positive selection and neutral selection are two possible scenarios for how HEAT repeats 9A and 10A may have evolved more drastically in the primate and murid lineages. While extensive phylogenetic and computational analyses of selective pressure in CRM1 failed to prove positive selection, we find it remarkable that these “bursts” of diversification within CRM1 HEAT repeats 9A and 10A were maintained over the subsequent 80 million years (Figures 6 and 7). Considering that this region of CRM1 clearly exhibits important biological relevance, at least to HIV-1, we suggest that positive, pathogen-driven selection may well underlie the emergence of these key CRM1 residues. We can only speculate as to the source of such selective pressure, but emphasize that modulation of nuclear membrane transport is critical for retroviral replication. For example, all lentiviruses such as HIV-1, as well as deltaretroviruses like HTLV, encode Rev-like proteins that use CRM1 to regulate the export of intron-containing RNA from the nucleus. Notably, neither lentiviruses nor deltaretroviruses are associated with natural infection of rodents. Despite this apparent exclusion, a Rev equivalent, Rem, was recently identified for the betaretrovirus mouse mammary tumor virus [51], [52], indicating that CRM1 may indeed be co-opted by rodent retroviruses.

In sum, we hypothesize that CRM1 was subject to a strong selection event in the primate lineage ∼80 million years ago that altered the sequence of HEAT repeat 9A. While we do not know the pathogen (or other selective pressure) that caused this, we have shown that the resulting CRM1 sequence is better able to support HIV-1 Rev's function as a mediator of viral RNA nuclear export. This may therefore serve as an example of the complexity of the pathogen-host “arms race”, wherein protein evolution in response to one pathogen has, over time, provided a useful foothold for the efficient replication of another.

## Materials and Methods

### Cell culture, plasmids and stable cell lines

Cells were cultured in Dulbecco's modified Eagle medium supplemented with 10% fetal bovine serum plus L-glutamine and penicillin/streptomycin. The pGP-RRE-, pGP-4×CTE-based vectors, pBC12/IL-2, pcRev, pBC12/mCycT1-Y261C-3HA and pluciferase have been described [18], [19], [27], [53]. The 4xCTE was a kind gift of Hans Georg Kräusslich [54]. Murine and human CRM1 cDNAs were obtained from Open Biosystems (Thermo Scientific) and cloned into pcDNA3.1 (Invitrogen). An amino-terminal triple-myc encoding epitope tag was added to CRM1, luciferase and GFP expressing vectors using a modified triple-myc pcDNA3.1 (Invitrogen)-based plasmid. myc epitope tagged mouse-human chimeric cDNAs and mutants thereof were generated by overlapping PCR and also cloned into the triple-myc vector. The Rev-minus HIV-1_NL4-3_ provirus was generated by replacing the EcoRI-NheI fragment of pNL4-3 with that of pNL4-3Rev-/4xMS2 [55], generating pNL4-3/Rev-. To generate the 3T3.GFP-mCRM1 and 3T3.GFP-hCRM1 cell lines, the GFP reading frame was fused to the mCRM1 and hCRM1 reading frames using overlapping PCR and these DNAs were subsequently subcloned into a retroviral vector [56] for expression from transcripts also carrying an internal ribosomal entry site (IRES) and encoding neomycin-resistance.

### Assembly and infectivity assays

Cell lines were plated at ∼30% confluency in 6 well dishes prior to transfection using FuGene 6 reagent (Roche) following the manufacturer's instructions and medium was replaced at 24 h post-transfection. Levels of p24^Gag^ in viral supernatants were measured by enzyme-linked immunosorbent assay (ELISA) (Perkin Elmer). Viral infectivity was gauged by adding filtered supernatants in the presence of 5 µg/ml polybrene to TZM-bl indicator cells [57] at ∼50% confluency and measuring the induced expression of ß-galactosidase at 24 h using the Galacto-Star system (Applied Biosystems). Immunoblot analyses were carried out as previously described [18]. Gag was detected using mouse anti-p24^Gag^ antiserum 24-2 (diluted 1∶1,000) [58], myc-tagged species using mouse anti-myc antiserum (9E10) [59], CRM1 using rabbit anti-CRM1 ab24189 antiserum (Abcam) and HSP90 using rabbit anti-HSP90 antiserum (Santa Cruz Biotechnologies) followed by anti-mouse or anti-rabbit secondary antibodies conjugated to infrared fluorophore IRDye800 (Li-Cor Biosciences) for quantitative immunoblotting. Anti-mouse secondary antibodies conjugated to horse radish peroxidase (Pierce) were used for detection of the myc-tagged CRM1 species. For Figures 5B and S2, the protease inhibitor saquinavir (NIH AIDS Research and Reference Reagent Program) was added at 1 µM at 24 h post-transfection.

### Metabolic labelling and northern blot analysis

Rates of translation were analyzed using [^35^S]methionine-cysteine metabolic labeling as previously described [18]. RNA isolation and northern blot analyses were as described [26] with minor modifications. For nuclear/cytoplasmic separation, 3T3 cells were lysed in 400 µl of cold, low salt NB buffer (50 mM Tris-HCL pH 8.0, 20 mM NaCl, 1.5 mM MgCl_2_, 0.5% NP-40) at ∼40 h post-transfection, held on ice for 5 min and then centrifuged at 500 × g to pellet nuclei. 200 µl of the cytoplasmic fraction was added to 600 µl RLT buffer (Qiagen) and vortexed vigorously. The nuclear pellet was washed twice in cold, low salt NB buffer, lysed in RLT buffer and spun through a Qiashredder column (Qiagen). The ^32^P-labelled random primed probes for northern analyses were generated using HIV-1_NL4-3_ nucleotides 8465-8892 or a β*-actin* PCR fragment [60].

### Microscopy

3T3 cells were plated on glass coverslips, transfected and processed as described [18]. myc-tagged proteins were detected using anti-myc antiserum (9E10) [59] and Gag using mouse monoclonal anti-p24^Gag^ antiserum (24-2; diluted 1∶1,000 in NGB) [58] and rabbit polyclonal anti-p17^Gag^ serum (UP595; diluted 1∶500 in NGB) [19], respectively, followed by goat anti-mouse-AlexFluo546 and goat anti-rabbit-AlexaFluo488 fluorescent secondary antibodies (Invitrogen). Cell nuclei were visualized by staining with 4′,6-diamidino-2-phenylindole (DAPI). Cells were visualized using laser scanning confocal imaging on a DM IRE2 microscope (Leica). Images were processed using LCS (Leica) and Openlab (Improvision) software packages.

### Phylogenetic analysis

CRM1 sequences were retrieved from NCBI and Ensembl. The phylogeny of 17 full-length (3213 nt) mammalian CRM1 sequences was reconstructed by maximum likelihood (ML) inference, under the general time reversible model of nucleotide substitutions, using the program FastTree 1.0 [61]. The species included in the analysis were human (*Homo sapiens*), chimpanzee (*Pan troglodytes*), gorilla (*Gorilla gorilla*), gibbon (*Nomascus leucogenys*), macaque (*Macaca mulatta*), marmoset (*Callithrix jacchus)*, mouse *(Mus musculus),* rat (*Rattus norvegicus*), rabbit (*Oryctolagus cuniculus)*, dog (*Canis lupus familiaris)*, panda (*Ailuropoda melanoleuca)*, horse (*Equus caballus)*, microbat (*Myotis lucifugus*), cow (*Bos Taurus)*, pig (*Sus scrofa)*, elephant (*Loxodonta Africana)*, opossum (*Monodelphis domestica)*. Local support values of the phylogenetic branches were calculated on the basis of 1000 replicates. Trees were edited using the program FigTree v1.3.1 (http://tree.bio.ed.ac.uk/software/figtree). The ancestral reconstruction of amino acid alteration along the CRM1 phylogeny was performed by maximum likelihood inference under the Whelan and Goldman empirical model, as implemented in the program codeML from the package PaML v3.14a [62]. The reconstructed ML phylogeny was fixed for all subsequent selection analyses. For sliding window analysis we used KaKs Calculator v2 [63], using the modified LPB option, a window of 30 bp and step size of 3 bp.

The chicken (*Gallus gallus*) and zebra fish (*Dani rerio*) sequences were obtained from Ensembl. In addition, we analysed several CRM1 sequences from Enembl or NCBI that are from low-coverage or preliminary assemblies/annotation and therefore did not have full length CRM1 sequences. These included the bushbaby (*Otolemur garnetti*), tarsier (*Tarsius syrichta*), baboon (*Papio hamadryas*), orangutan (*Pongo abelii*), pika (*Ochotona princeps*), squirrel (*Spermophilus tridecemlineatus*), kangaroo rat (*Dipodomys ordii*), armadillo (*Dasypus novemcinctus*) and platypus (*Ornithorhynchus anatinus*). To reconstruct the tarsier CRM1 sequence, we used the NCBI trace archive database to nearly complete the partial sequence obtained from Ensembl. This allowed a sequence that had only two amino acid gaps (position 112 and 117) to be assembled. As this sequence is identical to several Laurasiatheria CRM1 sequences (horse, cow, dog, panda) and has only 1 difference compared to the rabbit (at position 396), which is not conserved in other Rodentia or primate sequences, this is considered the ancestral Euarchontoglires sequence. The mouse lemur (*Microcebus murinus*) CRM1 sequence for HEAT repeat 9A was obtained from the NCBI trace archive database.

Several approaches were used to investigate the role of selection on the CRM1 gene during mammal evolution. First, evidence for codon-specific positive selection was sought using three different maximum likelihood tests implemented in the HyPhy package [64]: SLAC (Single Likelihood Ancestor Counting), FEL (Fixed Effect Likelihood) and REL (Random Effects Likelihood). Analyses were conducted under the Hasegawa-Kishino-Yano (HKY85) model of nucleotide substitution, and the MG94xHKY85 model of codon evolution. The M7 model (neutral model) and the M8 model (positive selection model) implemented in the program codeML were also fitted to the sequence alignment. Model M7 assumes a beta distribution for dN/dS over sites limited to the interval (0,1), providing a null hypothesis for testing positive selection. Model M8 adds an extra class of dN/dS over sites to M7, allowing dN/dS >1. A likelihood ratio test was used to test whether allowing individual sites to evolve under positive selection (*i.e*, M8) provided a significantly better fit to the data than the neutral model (*i.e.*, M7). In the latter analysis, codon frequencies were calculated under the F3x4 model.

Second, the branch-site test implemented in codeML was used to identify codons subjected to positive selection along specific branches of the phylogeny (‘foreground’ branches) [65]. The two foreground branches tested were the one supporting the higher primate lineage (red branch in Figure 7A) and the one supporting the rodent lineage (blue branch in Figure 7A). These two branches were selected on the basis of the excess of amino acid changes they exhibit compared to other internal branches. Two models were compared: (i) model A, in which the foreground branches may have different proportions of sites under neutral selection than the rest of the phylogeny (i.e. relaxed purifying selection), and (ii) model B, in which the foreground branches may also have a proportion of sites under positive selection. A likelihood ratio test was performed to estimate whether model B gave a significantly better fit to the data. Each test was performed on the full-length CRM1 alignment (3213 nt), the HEAT repeat helix 9A region only (codon positions 402 to 423; 66 nt) and the HEAT repeat 10A region only (codon positions 469 to 481; 51 nt).

## Supporting Information

Figure S1 Co-expression of mCyclinT1-Y261C and hCRM1 combine to substantially improve the production of infectious HIV-1 particles. (A) 3T3 cells were transfected as for Figure 4A with HIV-1 pNL4-3 proviral plasmid and plasmids encoding mCycT-Y261C (lanes 3-5) or a control plasmid encoding IL-2 (lane 2). At ∼48 h post-transfection, equal volumes of filtered supernatants were used to infect TZM reporter cells. Error bars represent the standard deviation for 3 independent transfections. (B) Relative viral infectivity for the 3T3 cells supernatant from (A), sample 5, compared to virus harvested from HeLa cells transfected with 0.2 µg HIV-1_NL4-3_ plasmid and the indicated plasmids. Infectivity was calculated as the ratio of β-galactosidase (β-gal) units from the TZM assay to pg of input p24^Gag^, and normalized to the HeLa control sample (lane 2). Supernatants from (A), samples 2 and 3, were excluded from this analysis due to the low levels of virus generated. Error bars represent the standard deviation for 3 independent transfections.
(TIF)

Figure S2 hCRM1 expression in mouse cells stimulates Gag assembly in a dose-dependent manner. (A) 3T3 cells were transfected with plasmids encoding protease-defective Gag (GP-D25A-RRE) and increasing amounts of CRM1 plasmid as indicated. p55^Gag^, CRM1 and HSP90 were detected by immunoblot and Gag assembly efficiency was measured based on a “release factor”: the ratio of VLP-associated p55^Gag^ to cell-associated p55^Gag^. Values represent the fold change in release factor relative to the luciferase control (lanes 5 and 10).
(TIF)

Figure S3 CRM1 HEAT repeat helices 9A and 10A are at least 19 Å from the NES binding site. Both the Rev NES (blue) and amino acids differing between mCRM1 and hCRM1 (shown in red) were modeled onto the mCRM1 structure (PDB ID: 3NBZ) based on references [35], [36]. Pymol was used to generate the figure.
(TIF)

Figure S4 Evidence for positive selection in specific regions of CRM1. (A) Sliding window analysis of aligned CRM1 coding sequences from mCRM1 and hCRM1, each compared to a common ancestor, the horse. Dotted line indicates dN/dS = 1. HEAT repeat 9A consists of residue 405-423. (B) Evidence for positive selection in hCRM1 and mCRM1 HEAT repeat helix 9A. Sliding window analysis of aligned CRM1 coding sequences from the indicated pair of species.
(TIF)

Table S1 Potential positively selected sites on the primate and rodent lineages. Candidate sites were identified using a branch site test in codeML using codons 402-423 or 469-481.
(DOC)
