# Supplementary figures and images for: Evolution of a Species-Specific Determinant within Human CRM1 that Regulates the Post-transcriptional Phases of HIV-1 Replication
Source: PLoS Pathog. 2011 Nov 17;7(11):e1002395. doi: 10.1371/journal.ppat.1002395 (PMC3219727; doi:10.1371/journal.ppat.1002395)

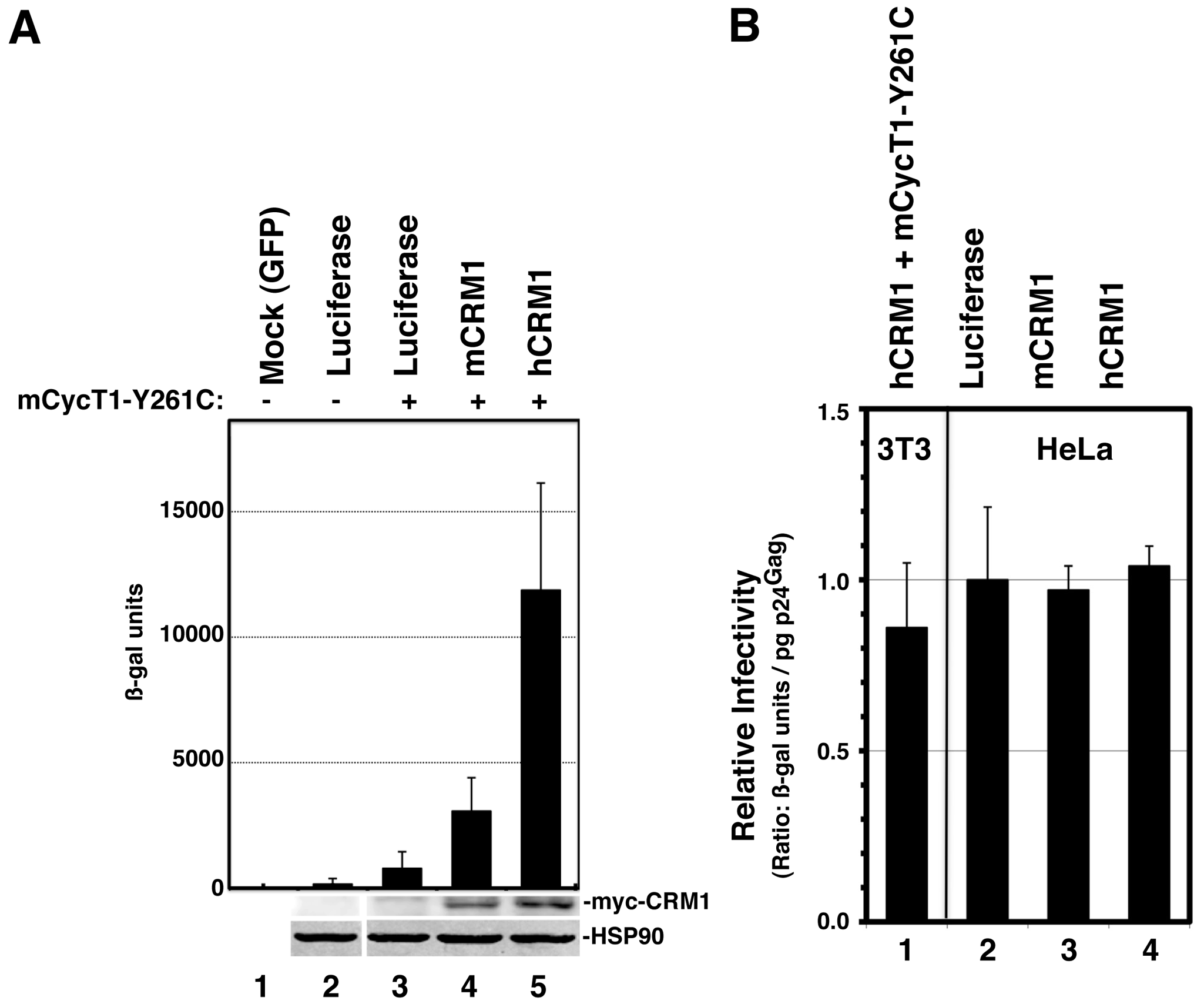

Supplement: Figure S1 — Co-expression of mCyclinT1-Y261C and hCRM1 combine to substantially improve the production of infectious HIV-1 particles. (A) 3T3 cells were transfected as for Figure 4A with HIV-1 pNL4-3 proviral plasmid and plasmids encoding mCycT-Y261C (lanes 3-5) or a control plasmid encoding IL-2 (lane 2). At ∼48 h post-transfection, equal volumes of filtered supernatants were used to infect TZM reporter cells. Error bars represent the standard deviation for 3 independent transfections. (B) Relative viral infectivity for the 3T3 cells supernatant from (A), sample 5, compared to virus harvested from HeLa cells transfected with 0.2 µg HIV-1NL4-3 plasmid and the indicated plasmids. Infectivity was calculated as the ratio of β-galactosidase (β-gal) units from the TZM assay to pg of input p24Gag, and normalized to the HeLa control sample (lane 2). Supernatants from (A), samples 2 and 3, were excluded from this analysis due to the low levels of virus generated. Error bars represent the standard deviation for 3 independent transfections. (TIF) [file ppat.1002395.s001.tif]

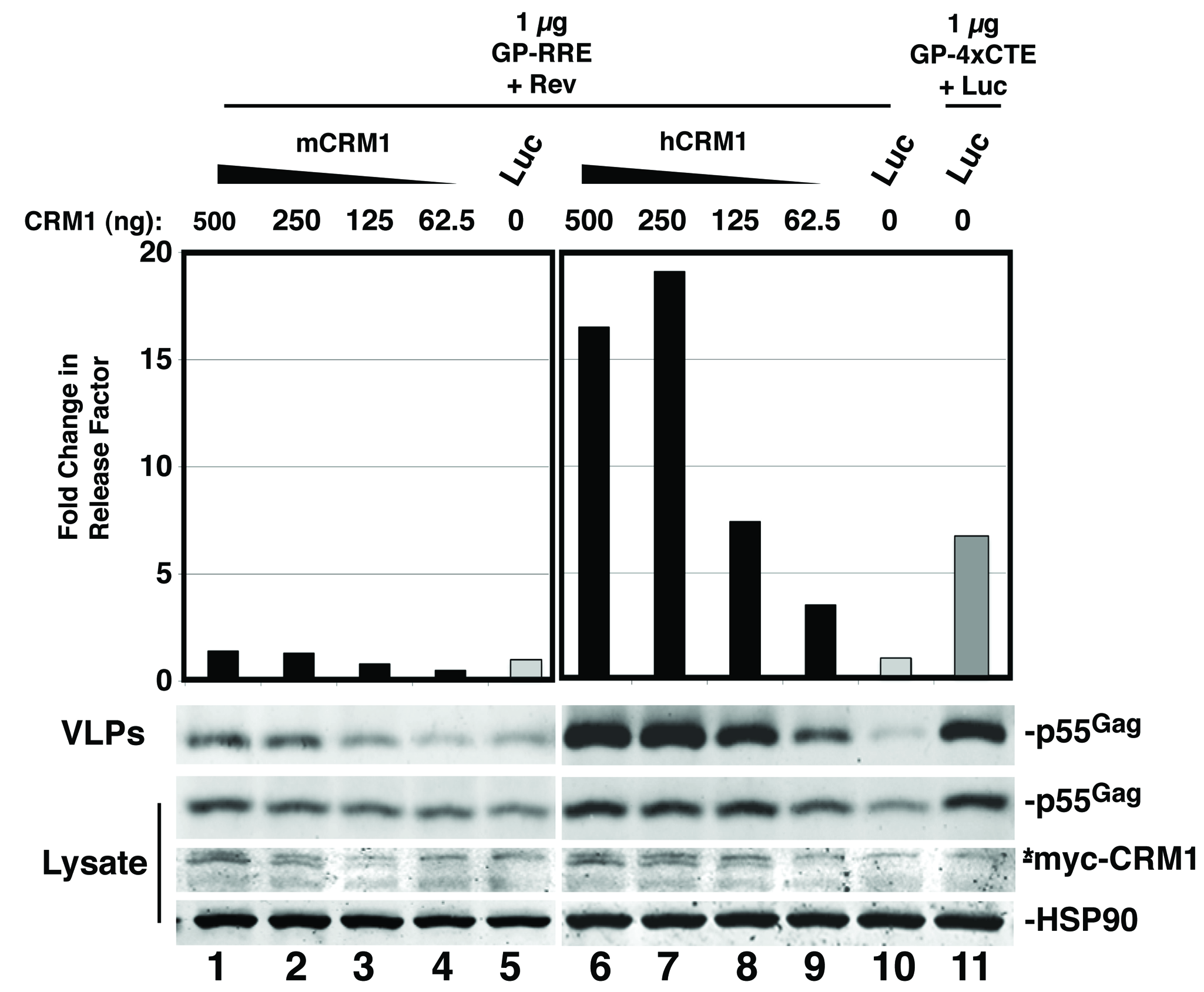

Supplement: Figure S2 — hCRM1 expression in mouse cells stimulates Gag assembly in a dose-dependent manner. (A) 3T3 cells were transfected with plasmids encoding protease-defective Gag (GP-D25A-RRE) and increasing amounts of CRM1 plasmid as indicated. p55Gag, CRM1 and HSP90 were detected by immunoblot and Gag assembly efficiency was measured based on a “release factor”: the ratio of VLP-associated p55Gag to cell-associated p55Gag. Values represent the fold change in release factor relative to the luciferase control (lanes 5 and 10). (TIF) [file ppat.1002395.s002.tif]

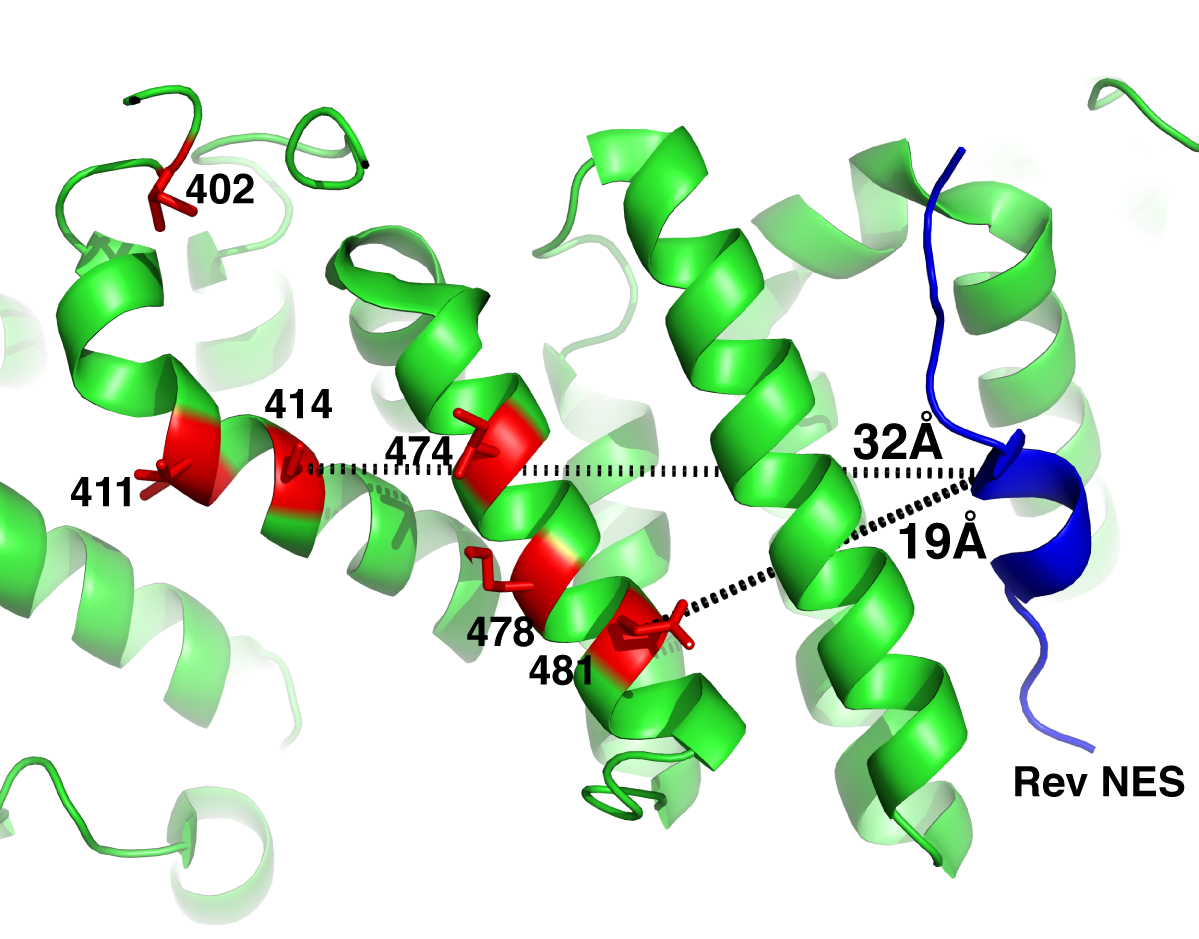

Supplement: Figure S3 — CRM1 HEAT repeat helices 9A and 10A are at least 19 Å from the NES binding site. Both the Rev NES (blue) and amino acids differing between mCRM1 and hCRM1 (shown in red) were modeled onto the mCRM1 structure (PDB ID: 3NBZ) based on references [35], [36]. Pymol was used to generate the figure. (TIF) [file ppat.1002395.s003.tif]

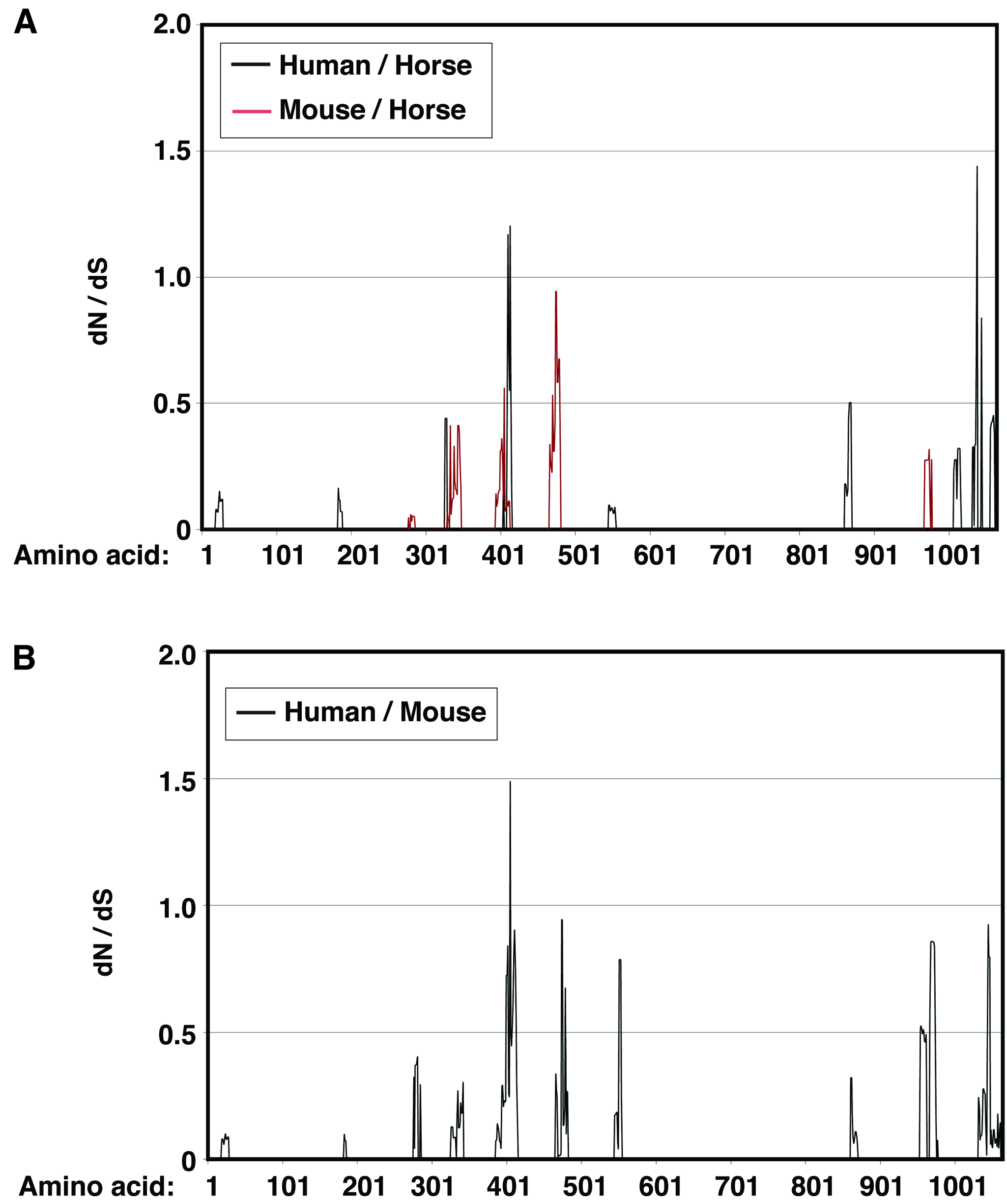

Supplement: Figure S4 — Evidence for positive selection in specific regions of CRM1. (A) Sliding window analysis of aligned CRM1 coding sequences from mCRM1 and hCRM1, each compared to a common ancestor, the horse. Dotted line indicates dN/dS = 1. HEAT repeat 9A consists of residue 405-423. (B) Evidence for positive selection in hCRM1 and mCRM1 HEAT repeat helix 9A. Sliding window analysis of aligned CRM1 coding sequences from the indicated pair of species. (TIF) [file ppat.1002395.s004.tif]
