# Supplementary material for: Evolution of a Species-Specific Determinant within Human CRM1 that Regulates the Post-transcriptional Phases of HIV-1 Replication
Source: PLoS Pathog. 2011 Nov 17;7(11):e1002395. doi: 10.1371/journal.ppat.1002395 (PMC3219727; doi:10.1371/journal.ppat.1002395)
Supplement: Table S1 — Potential positively selected sites on the primate and rodent lineages. Candidate sites were identified using a branch site test in codeML using codons 402-423 or 469-481. (DOC) [file ppat.1002395.s005.doc]

**Table S1**

| codon | prob(dN/ds)>1 |
| --- | --- |
| Primate branch | |
| 412 | 0.91 |
| 414 | 0.92 |
| Rodent branch | |
| 402 | 0.71 |
| 411 | 0.83 |
| 474 | 0.83 |
| 478 | 0.78 |
| 481 | 0.81 |
